# Safety and efficacy of risedronate for patients with esophageal varices and liver cirrhosis: a non-randomized clinical trial

**DOI:** 10.1038/s41598-019-55603-y

**Published:** 2019-12-12

**Authors:** Talles Bazeia Lima, Lívia Alves Amaral Santos, Hélio Rubens de Carvalho Nunes, Giovanni Faria Silva, Carlos Antonio Caramori, Xingshun Qi, Fernando Gomes Romeiro

**Affiliations:** 1 Internal Medicine Department, Gastroenterology Division – São Paulo State University (UNESP), Botucatu Medical School, São Paulo, Brazil; 2 Public Health Department, São Paulo State University (UNESP), Botucatu Medical School, São Paulo, Brazil; 3 General Hospital of Shenyang Military Command, Liaoning, Sheng China

**Keywords:** Liver cirrhosis, Portal hypertension

## Abstract

Despite the high prevalence of osteoporosis in liver cirrhosis, the indication of bisphosphonates for patients with esophageal varices has been avoided due to risk of digestive mucosal damage. Therefore, this study aimed to evaluate the safety profile of risedronate treatment for patients with osteoporosis, liver cirrhosis and esophageal varices with low risk of bleeding. A total of 120 patients were allocated into two groups according to their bone mineral density measured by dual-energy X-ray absorptiometry. In the intervention group, 57 subjects with osteoporosis received oral risedronate at 35 mg weekly plus daily calcium and vitamin D supplementation. In the control group, 63 subjects with osteopenia received only calcium and vitamin D. The groups received the treatment for one year and underwent surveillance endoscopies at six and 12 months, as well as a control dual-energy X-ray absorptiometry after a 12-month follow-up. The study received Institutional Review Board approval. The groups had not only comparable Model for End-stage Liver Disease score and esophageal varices degree, but also similar incidence of digestive adverse effects. A significant improvement was achieved in the intervention group in the lumbar spine T score (*p* < 0.001). The results suggest that risedronate may be safely used in liver cirrhosis and esophageal varices with low bleeding risk under endoscopic surveillance, thus allowing bone mass recovery.

## Introduction

Osteoporosis is a common complication of liver cirrhosis^1^ and leads to fractures that compromise quality of life and decrease survival^2,3^. The mechanisms involved include decrease in trophic factors^4^, increased levels of inflammatory mediators^5^, impairment of osteoblast differentiation and proliferation^6^, genetic polymorphisms^7^, hypogonadism^8^, alcoholism^9^, chronic corticosteroid use^10^ and viral hepatitis^11,12^. The relationship between the severity of liver disease and bone loss is not clear, and many patients with compensated cirrhosis have osteoporosis and fractures^13^. The dual-energy x-ray absorptiometry (DXA) has been applied to diagnose osteoporosis and should be performed in liver cirrhosis as soon as possible, especially in advanced liver disease, cholestatic disorders and chronic corticosteroids users^14^.

Osteoporosis treatment in cirrhotic patients includes adequate intake of calcium and vitamin D, physical activity and risk factors management, stopping tobacco and alcohol consumption and an anti-resorptive agent. Bisphosphonates are synthetic analogues of inorganic pyrophosphates with high affinity for mineralized tissues, thus inhibiting bone resorption. These drugs are associated with a reduced risk of bone fracture and mortality, both as intravenous^15^ and oral presentations^16^. However, intravenous infusions can cause undesirable reactions in up to one third of patients, leading to prolonged arthralgia and myalgia^17^, whereas oral bisphosphonates may cause digestive mucosal damage, causing dysphagia, esophagitis and ulcers^18^.

Such side effects have discouraged the prescription of oral bisphosphonates for patients with cirrhosis and portal hypertension, mainly due to the hypothetical risk of upper gastrointestinal hemorrhage arising from esophageal varices rupture^19^. However, this risk is probably overestimated^10^. In a randomized study on anti-osteoporosis medications for patients with viral hepatitis, bisphosphonates were the most effective. However, few subjects received alendronate and risedronate, some of them had no liver cirrhosis and the esophageal varices degree was not evaluated^20^. In another trial, patients with cirrhosis and osteoporosis were treated with ibandronate, but only 19 subjects completed the study and the follow-up was limited to six months^21^.

In view of the controversy and the lack of trials on oral bisphosphonate therapy in cirrhotic subjects, this study aimed to evaluate the safety of oral risedronate for patients with osteoporosis and liver cirrhosis with low-risk bleeding esophageal varices for one year. The secondary endpoint was the bone mineral density (BMD) recovery.

## Methods

### Patients

The study was a prospective, non-randomized and controlled trial approved by the local ethics committee (named “Comitê de Ética em Pesquisa” - protocol 089211-2013) at December, 19^th^ 2013. The subjects were attended the Hepatology units at UNESP Hospital (Botucatu, São Paulo state, Brazil).

The project was registered in the REBEC clinical trials platform (http://www.ensaiosclinicos.gov.br), which is a publically accessible primary International Clinical Trial Registry Platform. The Clinical trial registration number and the date of registration were RBR-76pm35 and 10/11/2015. The study was carried out according to the Declaration of Helsinki and its revisions.

The Outpatients aged more than 18 years with liver cirrhosis, esophageal varices and osteoporosis or osteopenia were included between March 2014 and February 2017. The diagnosis of cirrhosis was confirmed through data on liver biopsy or by associating radiological and endoscopic findings compatible with cirrhosis and portal hypertension. Informed consent was obtained from all participants. The exclusion criteria were creatinine clearance below 30 ml/min, upper gastrointestinal hemorrhage in the last two months, gastric varices without endoscopic treatment, active peptic ulcer, severe vascular ectasia, esophageal stricture, achalasia, gastroparesis, bisphosphonates hypersensitivity, liver transplantation, hormone replacement therapy and primary hyperparathyroidism. Pregnant or lactating women, patients with esophageal, gastric or duodenal neoplasms and those using non-steroidal anti-inflammatory drugs, anticoagulants or alcoholic beverages were also excluded. The patients addicted to alcohol were not allowed to participate because their alcohol consumption would make them more prone to developing complications, such as variceal bleeding.

Eligible patients were initially submitted to DXA (Discovery QDR Hologic, Inc) for assessing BMD at the lumbar spine and femoral neck. The results were expressed as the standard deviation in relation to the mean of the young population (T score), according to the World Health Organization^22^.

Those with normal DXA were excluded, while the ones who had osteoporosis or osteopenia were submitted to esophagogastroduodenoscopy. Individuals with large or medium esophageal varices with red wale marks were included only after being submitted to endoscopic variceal band ligation (EVBL), sequentially repeated every six-eight weeks until achieving a low-risk status, as follows: eradicated, small or medium esophageal varices without red wale marks. Then, the subjects were allocated into the intervention or the control group according to their BMD values.

### Intervention group

Subjects with osteoporosis received a weekly risedronate dose of 35 mg for one year, plus vitamin D (400 units orally twice daily) and calcium supplementation in order to reach the minimum value of 1000 mg/day (through the diet and/or calcium carbonate tablets, based on the daily calcium intake). Risedronate was chosen because its lower risk of gastric ulcers^23^. The subjects were instructed to take the tablet while fasting, with 250 ml of water and not lying down or eating for at least 30 minutes. Diet recommendations were provided during dietician consultations pre- and post-intervention, encouraging the patients to keep in contact with the dietitian throughout the trial by phone calls and/or additional appointments. The subjects’ adherence and the occurrence of adverse events were assessed by phone calls and at medical appointments scheduled trimonthly, with additional appointments before each esophagogastroduodenoscopy.

After the pre-intervention esophagogastroduodenoscopy and EVBL procedures when the risk of bleeding was high, each subject was submitted to endoscopic reassessments at the 6^th^ and 12^th^ month. If the esophageal varices had achieved a high-risk degree, with large or medium diameter with red wale marks, a new EVBL was performed, regardless of beta-blocker use. When EVBL was performed, the withdrawal of only one risedronate dose was warned, resuming the treatment after one week and scheduling a new esophagogastroduodenoscopy after six-eight weeks until achieving a low-risk status.

### Control group

Subjects with osteopenia were submitted to the same approach but did not receive risedronate. Since bone density does not interfere in risedronate safety, which was the primary endpoint of the trial, only patients with liver cirrhosis, esophageal varices and osteopenia composed the control group.

### Outcomes

The primary outcomes were the upper gastrointestinal hemorrhage incidence and endoscopic findings related to digestive mucosal damage potentially caused by risedronate. The incidence of other adverse events, such as severe portal hypertensive gastropathy, dyspeptic symptoms and musculoskeletal disorders were also documented. Only complaints within 48 hours of the treatment commencement that limited daily activities without any other causality (e.g. trauma, overload, infections) were considered^24^. The musculoskeletal complaints were divided into myalgia (muscle pain) and arthralgia (joint pain). The secondary endpoint was the BMD recovery.

### Endoscopic evaluation

The esophagogastroduodenoscopies were performed with Olympus gastroscope models GIF-Q150 and GIF-Q180. Validated classifications were applied for standardization of the endoscopic finding. Peptic ulcers, esophagitis, esophageal varices and portal hypertensive gastropathy were graded according to Sakita^25^, Los Angeles^26^, Japanese Research Society for Portal Hypertension classification^27^ and McCormack^28^ classifications, respectively. Gastritis was not graded because all the subjects had portal hypertensive gastropathy, impairing gastritis assessment through the Sydney classification^29^. Duodenitis was also not graded due to the lack of a specific endoscopic classification.

### Sample size calculation

The incidence of digestive damage in cirrhotic patients who do not use risedronate vary from 10 to 30% per year^30^. As there are no data on digestive damage among risedronate users with liver cirrhosis, the following data were considered to estimate the risk. Esophagitis is a predictor of variceal upper gastrointestinal hemorrhage after EVBL^30^. In addition, some drugs such as non-steroidal anti-inflammatory drugs double the risk of gastrointestinal bleeding on cirrhotic subjects^31^. Finally, in women without cirrhosis, bisphosphonates cause esophagitis and/or gastric lesions in 10 to 40% of patients^23^. Hence, it was estimated that risedronate could increase the upper digestive tract damage by 30%, requiring a sample of 116 individuals.

### Statistical analysis

Paired *t* test and Wilcoxon Signed Rank test were applied for assessing T score variation at the lumbar spine and femoral neck before and after treatment. Comparisons between the groups where done using *t* test for parametric variables and Mann-Whitney Rank Sum Test for non-parametric variables, whereas dichotomous events were compared through the Chi-square and Fisher exact tests. Cox multiple regression model was used for assessing relative risks. The significance level adopted was 5% and the softwares Sigmastat version 3.5 and SPSS v21.0 were used.

Multiple imputations were carried out to attribute values to the missing data. They were performed via the software R by using the mice package and the logreg method for binary outcomes. The stripplot graph function was applied to verify the adequacy of the imputations by calculating the mean difference between the observed and imputed incidences for each outcome^32^. All the authors had access to the study data, and the final version of the article was approved by all of them.

## Results

**Figure 1:**
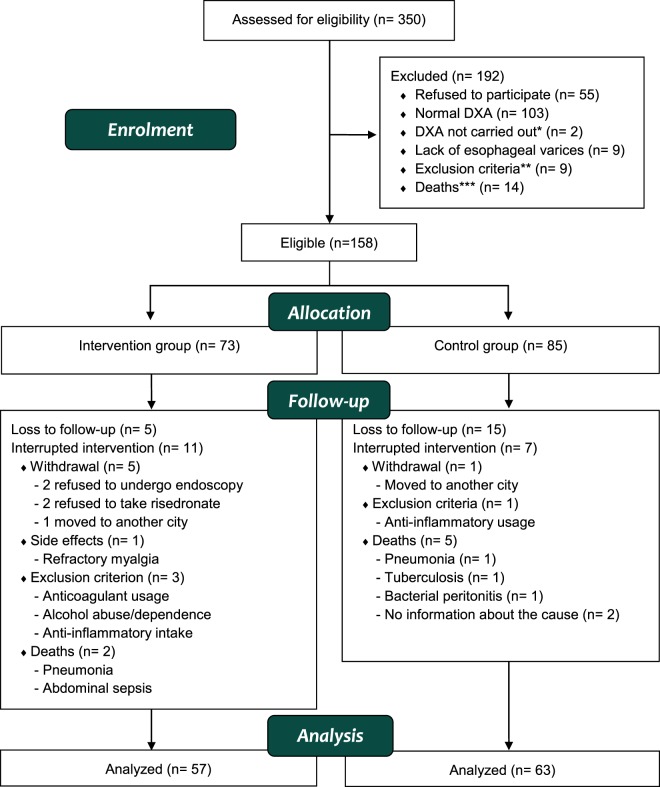
*DXA precluded because of excessive weight. **Alcohol abuse/dependence (n = 2), high-risk gastric varices (n = 3), active ulcer (n = 1), severe vascular ectasia (n = 1), creatinine clearance below 30 ml/min (n = 1), anticoagulant usage (n = 1). ***Pneumonia (n = 7), upper gastrointestinal hemorrhage (n = 1), lower gastrointestinal hemorrhage (n = 1), liver (n = 1) and head and neck (n = 1) cancer, endocarditis (n = 1), abdominal sepsis (n = 1), no informed cause of death (n = 1).

A total of 350 patients with liver cirrhosis were enrolled. The reasons that prevented the inclusion of some patients, the exclusions before and during the trial and the number of analyzed subjects are depicted in Fig. 1.

The total number of patients with or without esophageal varices who were submitted to DXA during the subjects’ enrolment was 283, with respective prevalence rates of osteoporosis and osteopenia of 28% and 35%.

### Baseline comparisons

The intervention group was older and had more women than the control group. In addition, alcoholic liver disease was more common than hepatitis C virus infection in the control group. Six subjects in the intervention group and one in the control group had had low-impact fractures before the study and four out of these seven individuals were chronic proton-pump inhibitors users. The other variables were similar between the groups (Table 1).

**Table 1:** Baseline comparisons of the groups.

Parameters	Intervention group (n = 57)	Control group (n = 63)	*p* value
Age in years (range)	61 (27–79)	55 (30–73)	0.008^a^
Gender (male/female)	26 (46%)/31 (54%)	48 (76%)/15 (24%)	0.003^b^
Caucasian ethnicity	50 (88%)	60 (95%)	0.149^c^
Cirrhosis etiology			
Hepatitis C	31 (54.4%)	23 (36.5%)	0.117^b^
Alcoholic liver disease	8 (14%)	24 (38%)	0.014^b^
Others	18 (31.6%)	16 (25%)	0.914^b^
Tobacco use	12 (21%)	14 (22%)	0.836^b^
Previous low impact fracture	6 (10%)	1 (2%)	0.096^c^
Acetylsalicylic acid use	1 (2%)	4 (6%)	1.000^c^
Prednisone use	4 (7%)	2 (3%)	0.664^b^
Child-Pugh Class A (%)	46 (81%)	44 (70%)	0.298^b^
Child-Pugh Class B (%)	10 (17%)	16 (25%)	0.609^b^
Child-Pugh Class C (%)	1 (2%)	3 (5%)	1.000^c^
MELD (range)	9.65 (6.43–15.53)	10.1 (6.43–19.07)	0.228^d^
Severe PHG	6 (11%)	6 (10%)	1.000^c^
Small and/or eradicated EV	50 (88%)	60 (95%)	0.149^c^
Medium EV	7 (12%)	3 (5%)	0.149^c^
Prior esophagitis	6 (11%)	5 (8%)	0.734^c^
Prior gastritis	17 (30%)	16 (25%)	0.659^b^
Prior ulcers	1 (2%)	3 (5%)	1.000^c^
Prior duodenitis	1 (2%)	4 (6%)	0.619^c^
Prior variceal UGIH	13 (23%)	22 (35%)	0.286^b^
Prior non-variceal UGIH	1 (2%)	0	0.480^c^
Prior EVBL	22 (39%)	29 (46%)	0.615^b^
Prior PPI use	36 (63%)	26 (41%)	0.042^b^
Prior beta-blocker use	30 (53%)	36 (57%)	0.717^b^

^a^T-test. ^b^Chi-square test. ^c^Fisher exact test. ^d^Mann-Whitney Rank Sum Test; MELD: model for end-stage liver disease; PHG: portal hypertensive gastropathy; EV: esophageal varices. UGIH: upper gastrointestinal hemorrhage; EVBL: endoscopic variceal band ligation; PPI: proton pump inhibitors. ^§^The less frequent etiologies of liver cirrhosis in the intervention and control groups were respectively: non-alcoholic fatty liver disease (6 and 4 cases), hepatitis B (3 and 6), autoimmune hepatitis (5 and 2), secondary biliary cirrhosis (3 and 0), hemochromatosis (1 and 1), cryptogenic cirrhosis (0 and 2), and progressive familial intrahepatic cholestasis (0 and 1).

### Endoscopic findings and adverse events

There were two cases of upper gastrointestinal hemorrhaging developed during the study, both in the control group and successfully treated through EVBL. The clinical and endoscopic characteristics of individuals with upper gastrointestinal hemorrhaging and peptic ulcers are described in Appendices 1 and 2, respectively. Four subjects in the intervention group and 10 individuals in the control group were submitted to EVBL during the study. Endoscopic findings were similar in both groups, but musculoskeletal pain was more frequent in the intervention group (Table 2).

**Table 2:** Incidence of endoscopic findings and adverse events in the one-year treatment.

	Intervention group (n = 57)	Control group (n = 63)	*p* value
UGIH	0	2	0.497^a^
Peptic lesions	26	31	0.694^a^
Ulcers	1	2	1.000^a^
Esophagitis	8	3	0.183^a^
Erosive gastritis	12	17	0.633^b^
Duodenitis	5	9	0.362^a^
Severe PHG	3	1	0.348^a^
EVBL	4	10	0.206^a^
Dyspeptic symptoms	11	4	0.063^a^
Musculoskeletal pain	8	1	0.047^b^
Arthralgia	4	1	0.343^a^
Myalgia	4	0	0.100^a^

^a^Fisher exact test. ^b^Chi-square test. UGIH: upper gastrointestinal hemorrhage; PHG: portal hypertensive gastropathy; EVBL: endoscopic variceal band ligation.

Eight cases of erosive esophagitis occurred in the intervention group and three cases in the control group. Most of them were grade A according to the Los Angeles classification. Dyspeptic symptoms were the most frequent complaint in both groups, controlled by correcting the risedronate administration, optimizing the proton-pump inhibitors’ dose or changing their class. Musculoskeletal pain was infrequent and most cases improved after painkiller prescription, but one patient was excluded due to refractory myalgia (Fig. 1).

### Bone analysis

**Figure 2:**
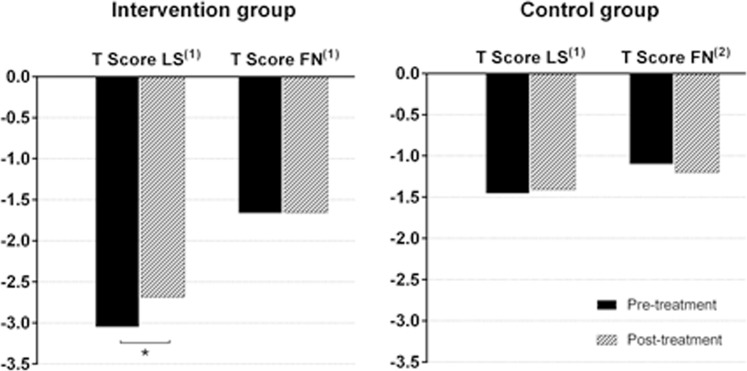
^(1)^Paired *t*-test. ^(2)^Wilcoxon signed rank test. *Statistically significant (*p* < 0.001). LS: lumbar spine. FN: femoral neck.

The mean T score at the lumbar spine of the intervention group increased from −3.053 to −2.674 (*p* < 0.001), with no significant improvement in the femoral neck values (*p* = 0.923). In the control group, no significant T score changes were observed, neither at the lumbar spine (*p* = 0.328) nor at the femoral neck (*p* = 0.304) (Fig. 2). There were no fractures related to osteoporosis during the follow-up.

### Risk analysis

**Figure 3:**
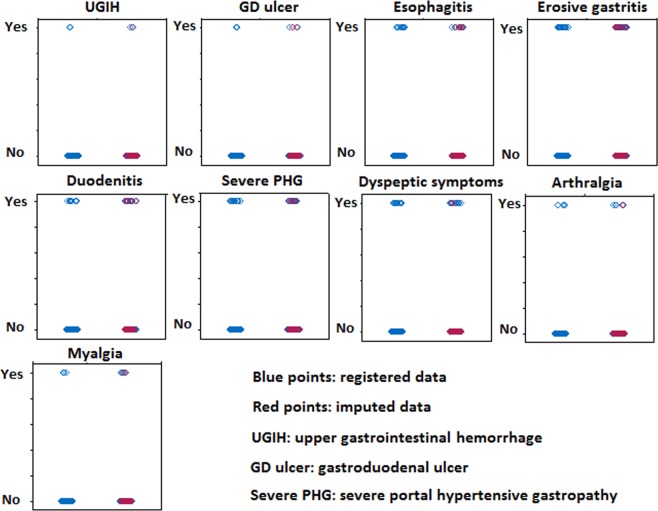
Multiple imputations performed to attribute values to the subjects who did not complete the study protocol. The imputations had a mean difference of 3.41% between the observed outcomes (blue) and the imputed ones (red). Each adverse event is described in two lines according to its presence (yes or no), and in two columns according to the inclusion or not of imputed data: the left columns show only the results from patients who complete the follow-up, while the right columns show results obtained by combining the observed data and the imputed ones.

Multiple imputations were performed to attribute values to the patients who had incomplete follow up (Fig. 3). A mean difference of 3.41% between the observed outcomes and the imputed ones was obtained, thus allowing calculation of the relative risks for each outcome in the intervention group (Table 3). The risks of digestive and musculoskeletal adverse events were low.

**Table 3:** Relative risks of each outcome in the intervention group compared to the control group.

Outcome	ARR	95% CI	p	RR	95% CI	p
UGIH	0.33	(0.007–14.95)	0.569	0.58	(0.05–6.42)	0.659
Peptic lesions	0.78	(0.44–1.38)	0.407	0.90	(0.55–1.48)	0.694
Ulcers	0.58	(0.07–4.31)	0.592	0.77	(0.13–4.64)	0.781
Esophagitis	1.49	(0.48–4.63)	0.489	1.49	(0.55–4.02)	0.423
Gastritis	0.67	(0.32–1.41)	0.291	0.77	(0.41–1.46)	0.433
Duodenitis	0.58	(0.80–1.66)	0.309	0.77	(0.31–1.90)	0.579
PHG	2.05	(0.84–5.02)	0.116	1.51	(0.66–3.45)	0.324
Dyspeptic symptoms	1.78	(0.56–5.66)	0.326	2.13	(0.79–5.77)	0.135
Musculoskeletal pain	1.24	(0.28–5.47)	0,777	1.74	(0.49–6.18)	0.388
Arthralgia	1.73	(0.29–10.09)	0.543	1.55	(0.34–6.93)	0.565
Myalgia	1.05	(0.14–7.94)	0.962	2.32	(0.42–12.71)	0.324

ARR: Adjusted relative risk of each outcome in the intervention group compared to the control group, adjusted for gender, age, ethnicity and prior UGIH, MELD score, gastric varices, PHG degree, esophagitis, gastritis, ulcer, duodenitis and degree of esophageal varices. RR: relative risk. 95% CI: 95% confidence interval. UGIH: upper gastrointestinal hemorrhage; PHG: portal hypertensive gastropathy; MELD: model for end-stage liver disease.

Combining the data obtained, the results suggest a favorable effect of risedronate treatment, with low risk of adverse events and significant bone improvement. Nonetheless, they were achieved in a strictly controlled sample, not allowing generalizations to any cirrhotic patient.

## Discussion

Data on bisphosphonates safety in liver cirrhosis are scarce and directed at assessing effectiveness^20,21^. This study suggests that patients with compensated cirrhosis and low-risk bleeding esophageal varices can take oral risedronate safely. There was no upper gastrointestinal hemorrhage, the incidence of endoscopic lesions attributable to the drug was low and the intervention led to BMD recovery. However, the results could not be extrapolated to patients with advanced liver disease and/or high-risk esophageal varices because most subjects had small and/or eradicated varices.

The intervention group was older and included more women than the control group. Likewise, proton-pump inhibitors consumption was also higher in the intervention group (*p* = 0.042). The mechanism by which these drugs may lead to bone mass reduction is not well understood, but the reduction in intestinal calcium absorption and a direct effect on BMD have been proposed^33^. A significant proportion of cirrhotic patients with esophageal varices can have abnormal gastroesophageal reflux^34^, thus increasing proton-pump inhibitors consumption. In addition to the risk of bone loss, these drugs also increase the risk of spontaneous bacterial peritonitis in advanced disease^35^.

Hepatitis C was the main liver disease in the intervention group, while alcoholic liver disease was the most frequent in the control group. Alcohol intake is an independent risk factor for bone loss^9^ and hip fracture risk is 5-fold higher in alcoholic liver cirrhosis^36^. However, comparing cirrhosis caused by alcohol versus hepatitis C virus, Carey *et al*. concluded that patients with this viral infection had lower BMD than ex-alcohol users^37^; furthermore, Mankal *et al*. found that alcoholic liver disease is associated with an increased risk of hepatic decompensation^38^.

The main risk factors for esophageal varices rupture are the portal pressure gradient and the presence of large vessels with red spots^39^. As the sample did not include patients with large esophageal varices and the control group had a higher prevalence of alcoholic liver disease, a higher portal pressure gradient would be expected in the control subjects, leading to two variceal upper gastrointestinal hemorrhages and the need for more EVBL, although the difference between the groups was not statistically significant.

The presence of esophageal varices was previously associated with reflux episodes, although it is unclear whether gastroesophageal reflux disease would increase the risk of rupture of varices^39^. Thus, it is also doubtful whether esophageal erosions caused by bisphosphonates could lead to a significant risk of upper gastrointestinal hemorrhage. The erosive esophagitis incidence did not differ between the groups, but only the intervention group had two cases of moderate and severe lesions.

The three individuals who had ulcers during the study were not infected by *H*. *pylori* and had no bleeding. In a prior study, gastric ulcer prevalence was significantly higher in patients with cirrhosis (20.8%) compared to non-cirrhotic patients (4%), whereas the only predictor observed was the portal pressure gradient^40^.

The difference between the groups in relation to dyspeptic symptoms did not reach the significance level. Only two out of the eight cases of esophagitis in the intervention group and one out of the three cases in the control group complained of dyspeptic symptoms. No relationship was found between dyspeptic complaints and the incidence of endoscopic findings.

Bisphosphonates are the most prescribed medications for osteoporosis^41^, but published studies on their side effects are scarce. Musculoskeletal pain is a common adverse event caused by these drugs and was more frequent in the intervention group, but most cases were easily controlled and only one subject discontinued the treatment due to severe myalgia, which ended after the drug’s withdrawal.

None of the seven deaths during the study were considered related to risedronate (most of them were caused by infections). Moreover, the improvement in bone density was clearly obtained owing to risedronate usage. This is the first time that such a finding was documented in a study composed only of cirrhotic patients, who are usually prevented from taking oral biphosphonates due to concerns that esophagitis could increase the risk of esophageal varices bleeding^13^.

Among the study limitations are the non-randomization and the double prophylaxis against variceal bleeding with beta-blockers and EVBL, while the current guidelines propose that only one of them should be performed as primary prophylaxis. Additional issues were the high dropout rate and the low adherence to treatment already reported among cirrhotic patients^42^. However, the dropout rates were 21.91% and 25.88% in the intervention and the control groups, respectively, with no significant difference between these proportions (p = 0.784). Furthermore, the trial had the largest sample size of cirrhotic patients using bisphosphonates while the multiple imputations enabled calculation of the relative risks to the subjects who received the intervention. The patients’ strictly controlled profile and the need for endoscopic follow-up may also be considered study limitations that could weaken the generalizability of the obtained data.

## Conclusions

The safety assessment of this trial suggests that oral risedronate can be used by patients with liver cirrhosis and low-risk esophageal varices under endoscopic surveillance, thus allowing BMD improvement at the lumbar spine.

## Supplementary information

Supplementary information

Research Protocol

## Data Availability

The datasets generated during and/or analyzed during the current study are available from the corresponding author on reasonable request.
