# Supplementary material for: Safety and efficacy of risedronate for patients with esophageal varices and liver cirrhosis: a non-randomized clinical trial
Source: Sci Rep. 2019 Dec 12;9:18958. doi: 10.1038/s41598-019-55603-y (PMC6908659; doi:10.1038/s41598-019-55603-y)
Supplement: Supplementary file 1 — Supplementary information [file 41598_2019_55603_MOESM1_ESM.docx]

Safety and efficacy of risedronate for patients with esophageal varices and liver cirrhosis: a non-randomized clinical trial

**Authors:** Talles Bazeia Lima*^1^; Lívia Alves Amaral Santos*^1^; Hélio Rubens de Carvalho Nunes^2^; Giovanni Faria Silva^1^; Carlos Antonio Caramori^1^; Xingshun Qi^3^ and Fernando Gomes Romeiro^1^.

*The first two authors contributed equally to this work (co-first authors).

^1^ Ph.D. – Internal Medicine Department, Gastroenterology Division – São Paulo State University (UNESP), Botucatu Medical School.

^2^ Ph.D. – Public Health Department – São Paulo State University (UNESP), Botucatu Medical School.

^3^ Ph.D. – General Hospital of Shenyang Military Command, Liaoning, Sheng, China.

Appendix 1. Characteristics of the two subjects with upper gastrointestinal hemorrhage

| **Variables** | **Subject 1** | **Subject 2** |
| --- | --- | --- |
| Group | Control | Control |
| Age | 39 | 68 |
| Sex | Female | Male |
| Ethnicity | Caucasian | Caucasian |
| Cirrhosis etiology | Cryptogenic | Hepatitis C |
| Tobacco use | No | No |
| Previous low impact fracture | No | No |
| Acetylsalicylic acid use | No | No |
| Prednisone use | No | No |
| Child-Pugh Class | B (8 points) | A (5 points) |
| MELD score | 15.28 | 7.5 |
| Prior PHG degree | Mild | Mild |
| Prior esophageal varices degree | Small | Small |
| Prior esophagitis | No | No |
| Prior gastritis | No | No |
| Prior ulcers | No | No |
| Prior duodenitis | No | No |
| Prior variceal UGIH | Yes | No |
| Prior non-variceal UGIH | No | No |
| Prior EVBL | Yes | No |
| Proton pump inhibitors PPI use | No | No |
| Beta-blocker use | Yes | No |

MELD: model for end-stage liver disease; PHG: portal hypertensive gastropathy; UGIH: upper gastrointestinal hemorrhage; EVBL: endoscopic variceal band ligation.

Appendix 2 - Characteristics of the three subjects with gastrointestinal ulcers

| **Variables** | **Subject 1** | **Subject 2** | **Subject 3** |
| --- | --- | --- | --- |
| Group | Intervention | Control | Control |
| Age | 63 | 68 | 53 |
| Sex | Male | Male | Male |
| Ethnicity | Caucasian | Caucasian | Caucasian |
| Cirrhosis etiology | Cryptogenic | Hepatitis C | Hepatitis C |
| Tobacco use | No | No | No |
| Previous low impact fracture | No | No | No |
| Acetylsalicylic acid use | Yes | No | No |
| Prednisone use | No | No | No |
| Child-Pugh Class | A (6 points) | A (5 points) | A (6 points) |
| MELD score | 7.4 | 7.5 | 10.11 |
| Ulcer location | Duodenum | Duodenum | Stomach |
| Sakita class | A1 | A2 | A2 |
| *Helicobacter pylori* infection | No | No | No |
| Prior PHG degree | Mild | Mild | Mild |
| Prior esophageal varices degree | Small | Small | Small |
| Prior esophagitis | No | No | No |
| Prior gastritis | No | No | Yes |
| Prior ulcers | No | No | No |
| Prior duodenitis | No | No | No |
| Prior variceal UGIH | No | No | No |
| Prior non-variceal UGIH | No | No | No |
| Prior EVBL | No | No | Yes |
| Proton pump inhibitors PPI use | Yes | No | No |
| Beta-blocker use | No | No | Yes |

MELD: model for end-stage liver disease; PHG: portal hypertensive gastropathy; UGIH: upper gastrointestinal hemorrhage; EVBL: endoscopic variceal band ligation.
