# Supplementary material for: Safety and efficacy of risedronate for patients with esophageal varices and liver cirrhosis: a non-randomized clinical trial
Source: Sci Rep. 2019 Dec 12;9:18958. doi: 10.1038/s41598-019-55603-y (PMC6908659; doi:10.1038/s41598-019-55603-y)
Supplement: Supplementary file 2 — Research Protocol [file 41598_2019_55603_MOESM2_ESM.docx]

**Research Protocol**

### Part 1

**Project summary**

Despite the high prevalence of osteoporosis in liver cirrhosis, the indication of bisphosphonates for patients with esophageal varices has been avoided due to risk of digestive mucosal damage. Therefore, this study aimed to evaluate the safety profile of risedronate treatment for patients with osteoporosis, liver cirrhosis and esophageal varices with low risk of bleeding. Methods: A total of 120 patients were allocated into two groups according to their bone mineral density measured by dual-energy X-ray absorptiometry. In the intervention group, 57 subjects with osteoporosis received oral risedronate at 35 mg weekly plus daily calcium and vitamin D supplementation. In the control group, 63 subjects with osteopenia received only calcium and vitamin D. The groups received the treatment for one year and underwent surveillance endoscopies at six and 12 months, as well as a control dual-energy X-ray absorptiometry after a 12-month follow-up. The study received Institutional Review Board approval (089211/2013). Results: The groups had comparable Model for End-stage Liver Disease score and esophageal varices degree. There was no upper gastrointestinal bleeding in the intervention group, but two cases in the control group. The groups did not differ as to the incidence of esophagitis and ulcers. Adverse events were infrequent, and a significant improvement was achieved in the intervention group in the lumbar spine T score (p < 0.001). Conclusions: The results suggest that risedronate can be safely used in liver cirrhosis and esophageal varices with low bleeding risk under endoscopic surveillance, thus allowing bone mass recovery.

**General information**

- Protocol title: Safety of risedronate for patients with esophageal varices and liver cirrhosis: a non-randomized clinical trial – 19/May/2019.
- Name and address of the sponsor/funder: São Paulo Research Foundation (FAPESP). Rua Pio XI, 1500 - Alto da Lapa – Zip code 05468-901 - São Paulo/SP – Brazil. Phone number: (+55) 11 3838 4000 Fax number: (+55) 11 3645 2421
- Name and title of the investigator who is responsible for conducting the research: Fernando Gomes Romeiro, MD; Ph.D. He was the investigator who designed the study, collected the data and drafted the manuscript. Address and telephone number of the research site: Internal Medicine Department, Gastroenterology Division – São Paulo State University (UNESP), Botucatu Medical School. Av. Prof. Mário Rubens Guimarães Montenegro, s/n. Distrito de Rubião Jr. s/n Botucatu – SP. Zip code 18618687
- Name and address of the clinical laboratory involved in the research: Botucatu Clinical Hospital. Av. Prof. Mário Rubens Guimarães Montenegro, s/n. Distrito de Rubião Jr. s/n Botucatu – SP. Zip code 18618687

**Rationale & background information**

In view of the controversy and the lack of trials on oral bisphosphonate therapy in liver cirrhosis, this study aimed to evaluate the safety of oral risedronate for patients with osteoporosis and liver cirrhosis with low-risk bleeding esophageal varices for one year. The secondary endpoint was the bone mineral density recovery.

**References (of literature cited in preceding sections)**

1. Yurci A, Kalkan AO, Ozbakir O, Karaman A, Torun E, Kula M, et al. Efficacy of different therapeutic regimens on hepatic osteodystrophy in chronic viral liver disease. Eur J Gastroenterol Hepatol. 2011;23(12):1206-12. DOI: <http://dx.doi.org/10.1097/MEG.0b013e32834cd6f6>
2. Bansal R, Kumar M, Sachdeva P, Kumar A. Prospective study of profile of hepatic osteodystrophy in patients with non-choleastatic liver cirrhosis and impact of bisphosphonate supplementation. United Eur Gastroenterol J. 2016;4(1):77-83. DOI: <http://dx.doi.org/10.1177/2050640615584535>
3. Lanza FL, Hunt RH, Thomson AB, Provenza JM, Blank MA. Endoscopic comparison of esophageal and gastroduodenal effects of risedronate and alendronate in postmenopausal women. Gastroenterology. 2000;119(3):631-8. DOI: <https://doi.org/10.1053/gast.2000.16517>
4. Otete H, Deleuran T, Fleming KM, Card T, Aithal GP, Jepsen P, et al. Hip fracture risk in patients with alcoholic cirrhosis: A population-based study using English and Danish data. J Hepatol. 2018;69(3):697-704. DOI: <http://dx.doi.org/10.1016/j.jhep.2018.04.002>
5. Yoshida H, Mamada Y, Taniai N, Yoshioka M, Hirakata A, Kawano Y, et al. Risk Factors for Bleeding Esophagogastric Varices. J Nippon Med Sch. 2013;80:252-9. DOI: <https://doi.org/10.1272/jnms.80.252>

**Study goals and objectives**

The study aimed to evaluate the safety profile of risedronate treatment for patients with osteoporosis, liver cirrhosis and esophageal varices with low risk of bleeding.

**Study Design**

The study was a prospective, non-randomized and controlled trial approved by the local ethics committee (protocol 089211-2013). The subjects were attended the Hepatology units at UNESP Hospital (Botucatu, São Paulo state, Brazil). The study received the Brazilian trial registration number RBR-76pm35 and was carried out according to the Declaration of Helsinki and its revisions.

**Methodology**

Outpatients aged more than 18 years with liver cirrhosis, esophageal varices and osteoporosis or osteopenia were included between March 2014 and February 2017. The liver cirrhosis diagnosis was confirmed through data on liver biopsy or by associating radiological and/or endoscopic findings compatible with liver cirrhosis and portal hypertension. Informed consent was obtained from all participants. The exclusion criteria were severe psychiatric illness, creatinine clearance below 30 ml/min, upper gastrointestinal bleeding in the last two months, gastric varices without endoscopic treatment, active peptic ulcer, severe vascular ectasia, oesophageal stricture, achalasia, gastroparesis, bisphosphonates hypersensitivity, liver transplantation, hormone replacement therapy and primary hyperparathyroidism. Pregnant or lactating women, patients with oesophageal, gastric or duodenal neoplasms and those using non-steroidal anti-inflammatory drugs (NSAIDs), anticoagulants or alcoholic beverages were also excluded. Eligible patients were initially submitted to dual-energy X-ray absorptiometry (Discovery QDR Hologic, Inc) for assessing bone mineral density at the lumbar spine and femoral neck. The results were expressed as the standard deviation in relation to the mean of the young population (T score), according to the World Health Organization.

Those with normal DXA were excluded, while the ones who had osteoporosis or osteopenia were submitted to esophagogastroduodenoscopy (EGD). Individuals with large or medium esophageal varices with red wale marks were included only after being submitted to endoscopic variceal band ligation (EVBL), sequentially repeated every six-eight weeks until achieving a low-risk status.

Subjects with osteoporosis received a weekly risedronate dose of 35 mg for one year, plus vitamin D (400 units orally twice daily) and calcium supplementation in order to reach the minimum value of 1000 mg/day (through the diet and/or calcium carbonate tablets, based on the daily calcium intake). Risedronate was chosen because it has been associated with a lower risk of gastric ulcers. The subjects were instructed to take the tablet while fasting, with 250 ml of water and not lying down or eating for at least 30 minutes. Diet recommendations were provided during dietician consultations pre- and post-intervention, encouraging them to keep in contact with the dietitian throughout the trial by phone calls and/or additional appointments. The subjects’ adherence and the occurrence of adverse events were assessed by phone calls and at medical appointments scheduled trimonthly, with additional appointments before each EGD.

After the pre-intervention EGD and the EVBL procedures when the risk of bleeding was high, each subject was submitted to endoscopic reassessments at the 6th and 12th month. If the EV had achieved a high-risk degree, with large or medium diameter with red wale marks, a new EVBL was performed, regardless of beta-blocker use. Although no major benefit has been demonstrated from a combination of beta-blockers and EVBL for primary prophylaxis, EVBL was done due to the lack of safety studies on the use of bisphosphonates in liver cirrhosis. When EVBL was performed, the withdrawal of only one risedronate dose was warned, resuming the treatment after one week and scheduling a new EGD after six-eight weeks until achieving a low-risk bleeding EV status.

Subjects with osteopenia were submitted to the same approach, but did not receive risedronate. Individuals with osteoporosis were not included in this group because they would be prevented from receiving the standard treatment. Since bone density does not interfere in risedronate safety, which was the primary endpoint of the trial, only patients with liver cirrhosis, esophageal varices and osteopenia composed the control group.

The primary outcomes were the upper gastrointestinal bleeding incidence and endoscopic findings related to digestive mucosal damage potentially caused by risedronate: peptic ulcers and esophagitis. The incidence of other adverse events, such as severe portal hypertensive gastropathy (PHG), dyspeptic symptoms and musculoskeletal disorders were also documented. The secondary endpoint was the bone mineral density recovery measured by the T scores before and after the treatment.

The EGDs were performed with Olympus gastroscope models GIF-Q150 and GIF-Q180. Validated classifications were applied for standardization of the endoscopic finding. Peptic ulcers, esophagitis, EV and PHG were graded according to Sakita, Los Angeles, Japanese Research Society for Portal Hypertension classification and McCormack classifications, respectively. Gastritis was not graded because all the subjects had PHG, impairing gastritis assessment through the Sydney classification.

Patients flowchart:

Excluded (n= 192)

♦  Refused to participate (n= 55)

♦  Normal DXA (n= 103)

♦  DXA not carried out* (n= 2)

♦  Lack of oesophageal varices (n= 9)

♦  Exclusion criteria** (n= 9)

♦  Deaths*** (n= 14)

Analyzed (n= 57)

Eligible (n=158)

Loss to follow-up (n= 5)

Interrupted intervention (n= 11)

♦ Withdrawal (n= 5)

- 2 refused to undergo endoscopy

- 2 refused to take risedronate

- 1 moved to another city

♦ Side effects (n= 1)

- Refractory myalgia

♦ Exclusion criterion (n= 3)

- Anticoagulant usage

- Alcohol abuse/dependence

- Anti-inflammatory intake

♦ Deaths (n= 2)

- Pneumonia

- Abdominal sepsis

Loss to follow-up (n= 15)

Interrupted intervention (n= 7)

♦ Withdrawal (n= 1)

- Moved to another city

♦ Exclusion criteria (n= 1)

- Anti-inflammatory usage

♦ Deaths (n= 5)

- Pneumonia (n= 1)

- Tuberculosis (n= 1)

- Bacterial peritonitis (n= 1)

- No information about the cause (n= 2)

Analyzed (n= 63)

## Analysis

Intervention group (n= 73)

Control group (n= 85)

## Allocation

## Follow-up

## Enrolment

Assessed for eligibility (n= 350)

**Safety Considerations**

The safety of research participants was ensured by medical appointments and by phone, encouraging the reporting of adverse events and maintaining the patients under a sctrict follow-up. Any adverse effects on individuals was registered.

**Follow-Up**

The research protocol provided medical appointments and phone calls as a follow up to the research participants for 12 months, when the research study was completed.

**Data Management and Statistical Analysis**

The incidence of digestive damage in cirrhotic patients who do not use risedronate vary from 10 to 30% per year. As there are no data on the digestive damage in LC among risedronate users, the following data were considered to estimate the risk. Esophagitis is a predictor of variceal upper gastrointestinal bleeding after EVBL. In addition, some drugs such as NSAIDs double the risk of gastrointestinal bleeding on liver cirrhosis. Finally, in women without LC, bisphosphonates cause esophagitis and/or gastric lesions detected in approximately 10 to 40% of patients. Hence, it was estimated that risedronate could increase the upper digestive tract damage by 30%, requiring a sample of 116 individuals.

Statistical analysis

Paired t test and Wilcoxon Signed Rank test were applied for assessing T score variation at the lumbar spine and femoral neck. Comparisons between the groups where done using t test for parametric variables and Mann-Whitney Rank Sum Test for non-parametric variables, whereas dichotomous events were compared through the Chi-square test and Fisher exact test. The significance level adopted was 5% and the software Sigmastat version 3.5 was used.

**Quality Assurance**

The protocol was carried out by professionals skilled in GCP, by whom the data were managed.

**Expected Outcomes of the Study**

It was expected that bisphosphonates were safe and that patients with liver cirrhosis and low-risk esophageal varices could be benefited after they received the medication.

**Dissemination of Results and Publication Policy**

Mr. Fernando Gomes Romeiro take the lead in publication.

**Duration of the Project**

Outpatients aged more than 18 years with liver cirrhosis, esophageal varices and osteoporosis or osteopenia were included between March 2014 and February 2017.

**Problems Anticipated**

Patients’ adherence to the treatment proposed.

**Project Management**

Talles Bazeia Lima, Lívia Alves Amaral Santos and Fernando Gomes Romeiro designed the study, conducted the patients, collected the data and drafted the manuscript. Hélio Rubens de Carvalho Nunes performed the statistical analyzes. Giovanni Faria Silva, Carlos Antonio Caramori, Xingshun Qi and Fernando Gomes Romeiro reviewed and modified the manuscript until achieving the final version of the article, which was approved by all the authors.

**Ethics**

The issue that was likely to raise ethical concerns was the risk of gastrointestinal bleeding during the trial, which was proven to be low. Informed consent was obtained from the research participants while medical appointments at the Hepatology units of the hospital.

**Informed Consent Forms**

The approved version of the protocol must have copies of informed consent forms (ICF), both in English and the local language in which they are going to be administered. However translations may be carried out after the English language ICF(s) have been approved by the ERC. If the research involves more than one group of individuals, for example healthcare users and healthcare providers, a separate specifically tailored informed consent form must be included for each group. This ensures that each group of participants will get the information they need to make an informed decision. For the same reason, each new intervention also requires a separate informed consent form

### Part 2

#### Budget

Lívia Alves Amaral Santos received a scholarship from São Paulo Research Foundation (FAPESP) – Grant 2014/22572-9. The total amount received was R$ 153,433.80 (USD 38,358.45). The scholarship was used to keep the researcher in full-time dedication to the project while she was developing the study, which corresponds to her Doctorade project.

Fernando Gomes Romeiro received a financial support from São Paulo Research Foundation (FAPESP) – Grant 2016/07117-9. The total amount received was R$ 104,278.60 (USD 26,069.65). This amount was used to buy a Olympus gastroscope model GIF-Q180, which was immediatelly donated to the Botucatu Clinical Hospital and has been used in the Endoscopy section. The hospital is a non-profitable institution that takes care of patients from a wide region in São Paulo state without costs to the patients, receiving only financial support from the state government.

#### Other support for the Project

None to declare.

#### Collaboration with other scientists or research institutions

The study was part of a collaboration plan that has been developed between Gastroenterologists from the São Paulo state University (UNESP) in Brazil and the General Hospital of Shenyang Military Command, Liaoning, Sheng, China. The collaboration has been headed by doctors Fernando Gomes Romeiro and Xingshun Qi, involving many students and health professionals from the two countries.

#### Links to other projects

None to declare.

#### Curriculum Vitae of investigators

The CV of the Principal investigator and each co-investigators should be provided. In general each CV should not be more than one page, unless a complete CV is specifically requested for.

#### Other research activities of the investigators

The Principal investigator should list all current research projects that he/she is involved in, the source of funding of those projects, the duration of those projects and the percentage of time spent on each.

#### Financing and Insurance

The financial support was only the ones received from São Paulo Research Foundation (FAPESP), as declared above. No insurance was provided.
